# Influence of impact speed on water droplet erosion of TiAl compared with Ti6Al4V

**DOI:** 10.1038/srep14182

**Published:** 2015-09-22

**Authors:** M.S. Mahdipoor, H.S. Kirols, D. Kevorkov, P. Jedrzejowski, M. Medraj

**Affiliations:** 1 Department of Mechanical and Industrial Engineering, Concordia University, 1455 de Maisonneuve Boulevard West, QC, Montreal, Canada H3G 1M8; 2 Rolls-Royce Canada Ltd. Energy, 9545 Cote-de-Liesse, Dorval, QC, Canada H9P 1A5; 3 Department of Mechanical and Materials Engineering, Masdar Institute, Masdar City, Abu Dhabi, UAE, P.O. Box 54224

## Abstract

Water Droplet Erosion (WDE) as a material degradation phenomenon has been a concern in power generation industries for decades. Steam turbine blades and the compressor blades of gas turbines that use water injection usually suffer from WDE. The present work focuses on studying erosion resistance of TiAl as a potential alloy for turbine blades compared to Ti6Al4V, a frequently used blade alloy. Their erosion behaviour is investigated at different droplet impact speeds to determine the relation between erosion performance and impact speed. It is found that the relationship is governed by a power law equation, ER ~ V^n^, where the speed exponent is 7–9 for Ti6Al4V and 11–13 for TiAl. There is a contrast between the observed speed exponent in this work and the ones reported in the literature for Ti6Al4V. It is attributed to the different erosion setups and impingement conditions such as different droplet sizes. To verify this, the erosion experiments were performed at two different droplet sizes, 464 and 603 μm. TiAl showed superior erosion resistance in all erosion conditions; however, its erosion performance exhibits higher sensitivity to the impact speed compared to Ti6Al4V. It means that aggressive erosion conditions decrease the WDE resistance superiority of TiAl.

Water droplet erosion (WDE) is a result of repetitive high-speed water droplet impacts on solid surfaces which generate impulsive pressure^1^. This impact wear phenomenon seen in high-speed moving components in environments containing water droplets has always been a challenge, as it may decrease their service life. For instance, water is sprayed to cool down the air intake to gas turbine compressors^2^. As a result, high speed impingements between the applied water droplets and the compressor’s rotating blades cause WDE, especially for their leading edges. Moreover, the water droplets form at the low pressure stages of steam turbines due to steam condensation, which results in erosion of the blades^1^,^2^. The erosion of these components reduces, to a great extent, the power generation efficiency of turbines^2^. Water impingement erosion can also be observed in aircraft’s aerofoils, missiles and helicopter rotors which is called rain erosion^1^. WDE is a complicated combination of several phenomena. The imposed normal pressure, the subsequent stress waves and the formed outflow jets are the main reasons for damage in the target material^1^,^3^. The magnitude of impact pressure, area exposed to stresses and time duration of each impact pulse are functions of impingement conditions. The velocity of outflow jets and subsequent stresses also depend on the impact conditions^4^. Hence, determining theoretical and empirical relationships between the erosion rate or erosion resistance (reciprocal of material loss rate) and the WDE test parameters including impact speed and droplet size have been attempted by several scientists^3^,^4^,^5^.

Theories based on the normal impact stresses, stress waves, and energy transferred to the target were proposed in the literature for WDE^3^,^4^,^5^. The impact speed is the main parameter considered in all of them. It was also confirmed by experimental results^6^,^7^,^8^,^9^,^10^ that the erosion performance and the damage mechanism change significantly at different impact speeds. Experimental data were fitted to different functional forms in order to find the relationship between the erosion performance (erosion rate) and the impact speed. The most important ones were summarized by Heymann^4^ and are listed below:


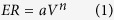



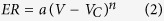



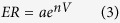


where *ER* is the erosion rate, *V* is the impact speed, *V*_*C*_ represents the threshold speed and *a* is a constant. The simple power relationship, equation (1), is the most used form to correlate *ER* and *V*^4^,^6^,^7^. However, it implies that WDE takes place regardless how low the impact speed is. Whilst, the common thought is that there is a critical or threshold speed called *V*_*C*_, below which erosion does not take place. Therefore, an erosion-impact speed relationship based on this concern was developed, equation (2)^4^, to fit the experimental data. Nevertheless, it was not as accurately representing the experimental results as equation (1) in the usual range of impact speeds (1.5 < V/V_C_ < 3). Another relationship based on the analogy of fatigue is equation (3); however, it was not referred to in the literature as much as the first two equations^4^,^11^. The speed exponent in equations (1) and (2) is an important value in the erosion performance investigations. It was claimed that the erosion damage is proportional to *V*^*2*^ for solid particle erosion^7^; however, it is not the case for water droplet erosion. A list of the reported speed exponent (*n*) values for different materials and their test conditions are summarized in Table 1. It is worth mentioning that the way of representing the *ER*_*max*_ is different in each case as explained in the table.

Clearly, studying the influence of impact speed on water erosion was not carried out based on a standard method. Its influence has been studied using different erosion indicators. Some research groups^9^,^12^ compared the average erosion rate at a specific exposure time, for example 30 hours. This type of comparison can be questionable because after 30 hours different samples might be at different erosion stages when tested at different speeds. Studying the relationship between impact speed and maximum erosion rate, Thirwvengadam’s work^6^, eliminates this concern. Moreover, with the large number of available experimental data, it was concluded that even the best proposed erosion rate-impact speed relationship would only fit the data over a limited speed range^4^,^11^. Indeed, this relationship is a function of water droplet erosion conditions and target material properties. The observed speed exponents for erosion rate-impact speed relationship, which can be seen in Table 1, are different because of diverse erosion conditions. Considering all of these studies^6^,^7^,^8^,^9^,^10^,^11^,^12^, it seems that using a standard method to find a dimensionless maximum erosion rate and determine its relationship with impact speed is necessary. This approach is proposed by ASTM G73 standard^13^ and is used in this study to calculate dimensionless erosion rate, unlike the previous works^6^,^7^,^9^,^10^.

Droplet size and its shape also affect the erosion damage. Unlike impact speed, the magnitude of impact stress was defined as independent of the droplet size or shape^3^,^4^,^5^. However, their influence on the erosion damage was observed in several experimental studies^8^,^11^,^14^,^15^,^16^. Honegger^8^ carried out erosion experiments using 0.5 and 1.5 mm jets and they found significant difference in the erosion damage. The difference was function of the utilized impact speed. Indeed, at higher impact speeds, the influence of water jet diameter on the erosion damage decreases. The erosion damage caused by six different jet diameters ranging from 1 to 2.5 mm was compared in another work^11^. For the jet diameters less than 1.6 mm, incubation period increased considerably by decreasing jet diameter. The influence of jet diameter on the incubation period was confirmed also by Hancox and Brunton for jets smaller than 1 mm^17^. It was demonstrated^4^ that threshold speed which corresponds to the erosion endurance was influenced by jet diameter based on the following relationship.


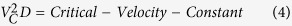


Heymann suggested that equation (4) can be generalized for water droplet erosion^4^; however, it was not verified experimentally. Decorso *et al.*^14^ confirmed that the larger the water droplets, the lower the threshold speed. They concluded that for the equal volume of impacting water, droplet size considerably affects the erosion damage for impact speeds close to the threshold. However, it does not influence the erosion when erosion tests are carried out at the impact speeds well above Vc. In the case of small droplets, the higher V_C_ and longer incubation period were attributed to the attenuation of impact stresses, less energy transferred to the target and lower chance for large fragment detachment^4^. Despite the mentioned explanation, there is no complete agreement on the reasons for the influence of water droplet shape and diameter. Recently, Hattori *et al.*^15^ and Ahmad *et al.*^16^ reported that the erosion rates of Al and Ti6Al4V are found to be proportional to the droplet diameter as, *Er* ∝ *V*^*4.7*^ for Al and *Er* ∝ *V*^*2.5*^ for Ti6Al4V. It is a notable dependency, which cannot be justified only by different duration of impact pulse or area exposed to the impacts. Therefore, more investigations need to be carried out in order to clarify the role of droplet size on the erosion damage.

Additionally, target material characteristics are effective parameters for water droplet erosion damage. Hardness, yield and ultimate strength, strain energy, modulus of resilience, hardenability, and crack-growth rate as a function of stress intensity (Paris law) are the mechanical properties of target material which were found to play roles in erosion damage^1^,^11^,^17^. None of these parameters has been accepted as the only index for erosion performance. Hardness is the most common property to which difference in erosion performance of materials relate. Heymann^11^ reported that erosion resistance of metals is proportional to the target hardness as, ∝*HV*^*2.5*^. He did not present direct relationship between strain energy or modulus of resilience and erosion resistance. An empirical value, which is the product of strength squared and modulus of elasticity, σ_u_^2^E, showed the best correlation with the WDE performance among the mentioned mechanical properties. Heymann^11^ demonstrated that WDE resistance is proportional to (σ_u_^2^E)^2/3^. Hence, the higher the strength and modulus of elasticity, the higher the erosion resistance. Such values can be assumed as primary indications for the erosion performance. However, to study the WDE behaviour, measuring the erosion resistance by itself is essential.

Over the years, TiAl alloys have been considered as suitable materials for aeroengine applications, such as engine vanes or blades^18^. Their high specific strength, modulus of elasticity, hardness, fatigue strength, and hardenability make them potential candidates for wear and erosion applications. These alloys were investigated mostly for high temperature applications^18^,^19^. However, some promising results about their cavitation erosion behaviour at room temperature were reported^20^,^21^. Nakao *et al.*^20^ investigated the cavitation erosion of different TiAl-based alloys compared to pure titanium and austenitic stainless steel. They reported that TiAl showed 20–30 times better cavitation erosion resistance than the other alloys. Howard and Ball^21^ attributed the high cavitation erosion resistance of TiAl alloys to their initial high tensile strength and high work hardening rate, compared to 304 stainless steel and WC-Co. High strain hardening of TiAl works as a surface treatment and increases the hardness upon water droplet impact which is beneficial for erosion performance. They also proposed that the erosion mechanism is based on the “twinning deformation” that happened in this alloy. Twinning at the tip of large cracks impedes their propagation and causes the formation of micro-cracks instead^22^. Such behaviour results in the decrease of crack growth rate, and it was mentioned as another reason for superior cavitation erosion performance of TiAl alloy^21^. Thereby, if twinning occurs upon water droplet impact, a deceleration in crack growth rate is expected, which is beneficial for WDE performance. Despite the great potential of TiAl alloy to combat WDE, their water droplet erosion behaviour could not be found in the literature.

The main objective of this work is to study the WDE behaviour of TiAl compared to Ti6Al4V. This has been accomplished through the following sections. First, the erosion performance (incubation period and maximum erosion rate) of TiAl and Ti6Al4V alloys at different impingement conditions are compared and their differences are justified based on the mechanical properties and microstructural changes. Secondly, the erosion rate-impact speed relationships for TiAl and Ti6Al4V, which is referred to in this article as Ti64, alloys are determined. Finally, the threshold speed or the erosion endurance of TiAl alloy for different droplets sizes is determined.

## Results

### Surface hardness and roughness of erosion test coupons

The surface roughness and hardness of prepared coupons were measured after polishing. Respectively, 0.082 ± 0.007 and 0.078 ± 0.004 μm are the surface roughness of Ti64 and TiAl. Although they were polished using 600 grit SiC grinding papers, small variation are observed for the values of surface roughness. The hardness of Ti64 and TiAl was found to be 284 and 338 HV10, these values are the averages of 5 readings. The hardness measurements were close and standard deviations were 12.5 and 9.1 for Ti64 and TiAl, respectively.

### Droplet size distribution

**Figure 1:**
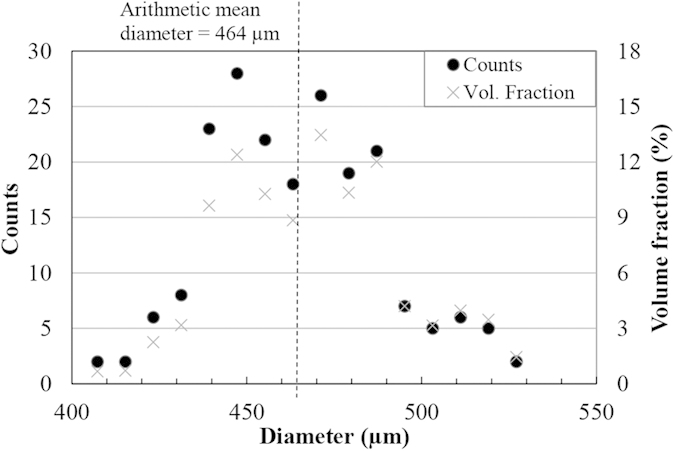
The number and volume droplet size distributions of 200 droplets generated using nozzle 1.

**Figure 2:**
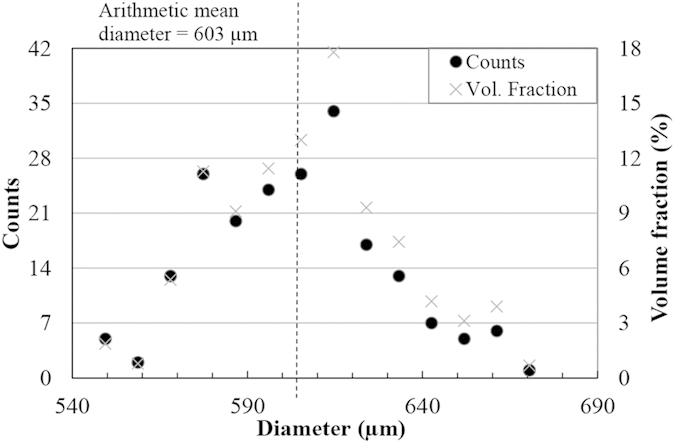
The number and volume droplet size distributions of 200 droplets generated using nozzle 2.

The diameters of 200 water droplets of each size were measured using a high speed camera and their distributions are presented in Figs 1 and 2.

Based on the droplet size distribution, the diameter of 78 percent of droplets range from 440 to 490 μm in case of nozzle 1 and the diameter of over 80 percent of droplets range from 575 to 635 μm for nozzle 2. Also, the presented volume fractions verify that a large volume of water (more than 75 percent) impacts the coupons as the droplets with diameter in the range of 430 to 490 μm and 575 to 635 μm. The arithmetic mean diameter based on the number of droplet counts was calculated as 464 μm for droplets generated by nozzle 1 and 603 μm for the ones generated by nozzle 2. To address droplet size further, these mean diameters are used.

### Water droplet erosion performance of TiAl and Ti64 alloys

#### Cumulative erosion, incubation period and maximum erosion rate

The erosion results are reported based on the cumulative material loss versus cumulative exposure. The cumulative material loss is defined as the difference in volume between the as-received specimen and the eroded specimen. The cumulative exposure can be represented using different parameters such as erosion time, number of impingements, and volume of water impacting the coupon^6^,^7^,^8^,^9^. In this study, the cumulative volume of water impinging the surface was used to represent exposure, which results in a dimensionless erosion rate. It is noteworthy that in most of previous studies^6^,^9^,^10^ either the number of rotations (in rotating arm designs) or exposure time (hours or minutes) were reported as the cumulative exposure parameter. These do not accurately describe WDE, since in most of these studies, the actual amount of water droplets impacting the samples was not known.

**Figure 3:**
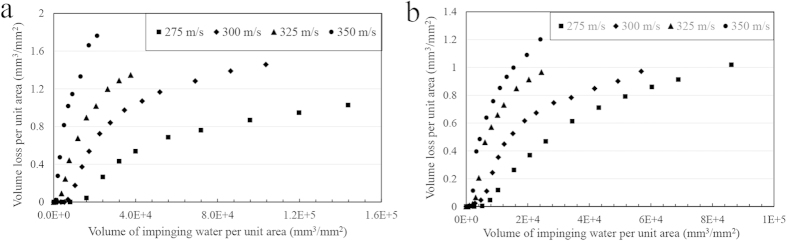
Water droplet erosion results, material loss versus volume of impinging water, for Ti64 specimens tested at different impact speeds and droplet size of (a) 464 μm, (b) 603 μm.

**Figure 4:**
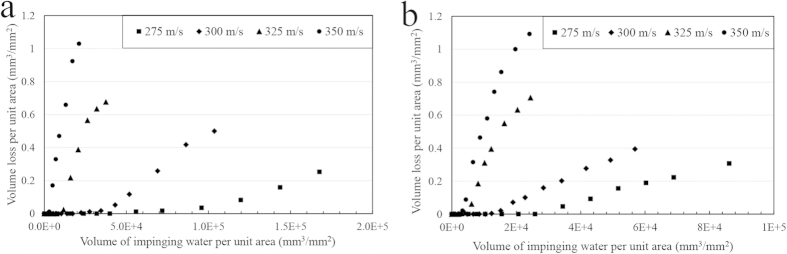
Water droplet erosion results, material loss versus volume of impinging water, for TiAl specimens tested at different impact speeds and droplet size of (a) 464 μm, (b) 603 μm.

The erosion test results of the current study are presented in Figs 3 and 4. They are plotted as the volume loss of material per unit area (mm^3^/mm^2^) versus the volume of impacting water droplets per unit area (mm^3^/mm^2^). In each figure, the erosion behaviour at constant droplet size but different velocities is demonstrated. Different stages of erosion damage can be easily identified in the plotted graphs of Ti64. However, this is not the case for TiAl alloy at low impact speeds and small droplets where erosion did not reach the terminal stage as shown in Fig. 4-a.

**Figure 5:**
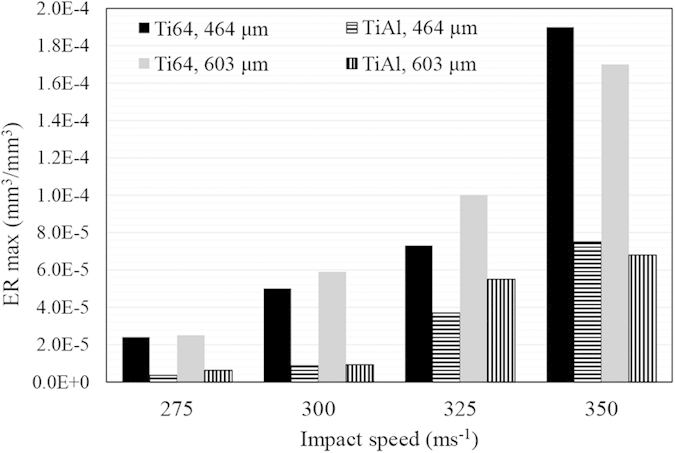
Maximum erosion rate of Ti64 and TiAl alloys eroded by 464 and 603 μm water droplets at four different impact speeds.

**Figure 6:**
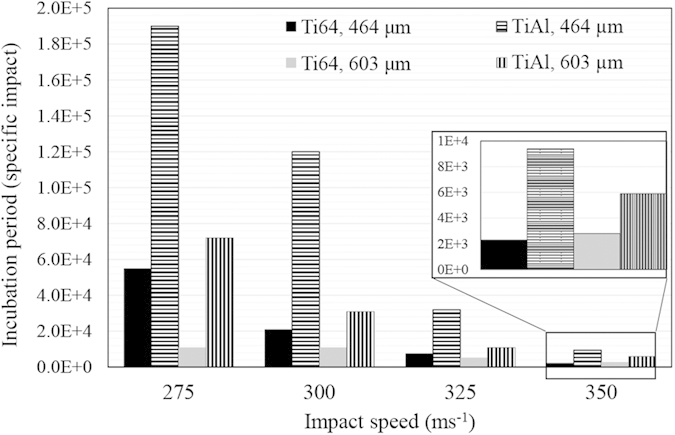
Incubation period of Ti64 and TiAl alloys eroded by 464 and 603 μm water droplets at four different impact speeds.

Different trends were observed for Ti64 and TiAl alloys’ erosion performances with changing the impact speed. The general propensity is that the higher the impact speed, the more the erosion damage. Furthermore, the larger the droplets size, the less the incubation period and the higher the maximum erosion rate. This is more evident for the erosion of Ti64. Here, erosion performance is represented by the incubation period (in terms of specific impacts), and the maximum erosion rate. These values were determined and plotted in Figs 5 and 6. TiAl shows 6 times lower *ER*_*max*_ than Ti64 and more than 3 times longer incubation at the least severe erosion condition. In case of the most aggressive erosion condition, it exhibits 2.5 times lower *ER*_*max*_ than Ti64 and only 2 times longer incubation period.

#### Influence of impact speed on incubation period and erosion rate

Increasing the impact speed results in increasing the localized impulsive pressure on the target^1^,^5^. The pressure resulting from liquid-solid impingement, known as the water hammer pressure, was firstly explained based on one-dimensional liquid-solid impact model^5^.


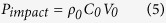


Where *ρ*_*0*_ is the liquid density, *C*_*0*_ represents the speed of sound in the liquid and *V*_*0*_ is the impact speed. It is a simplified condition which is not an accurate representation of reality. Heymann^3^ theoretically analyzed and proposed a more accurate model to include the effect of shockwave formation.


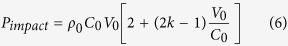


where *k* is an impinging liquid constant. The impact pressure is presented independent of the droplet size. In this study, Heymann’s equation is used to calculate the impact pressure corresponding to different erosion conditions. In the case of water droplet erosion *k, ρ* and *C* are 2, 1,000 kg/m^3^ and 1,463 ms^−13^, respectively.

In addition, the time duration of an impact pressure pulse was found to be a function of droplet diameter and equation (7) was introduced by Bowden and Field^23^.


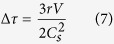


where *r* represents the radius of the water droplet front curvature, and *Cs* is the shock wave velocity in the water droplet. Table 2 presents the impact pressure and length of each impact pulse which help to explain different erosion performances.

**Figure 7:**
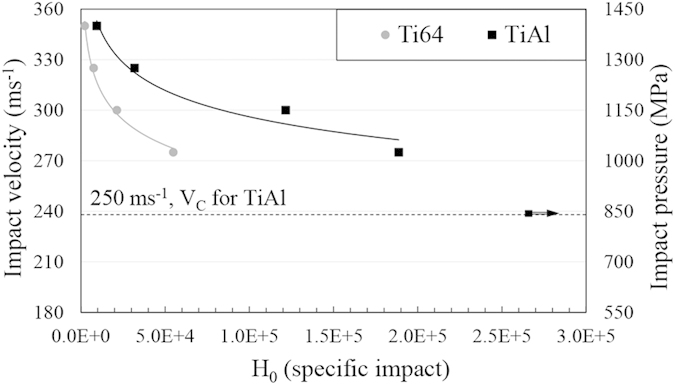
The dependency of Ti64 and TiAl incubation period on the impact speeds when they are eroded by 464 μm water droplets. The arrow indicates the test performed at 250 ms^−1^ and caused no measurable erosion after 10^6^ impact per 1 mm^2^.

**Figure 8:**
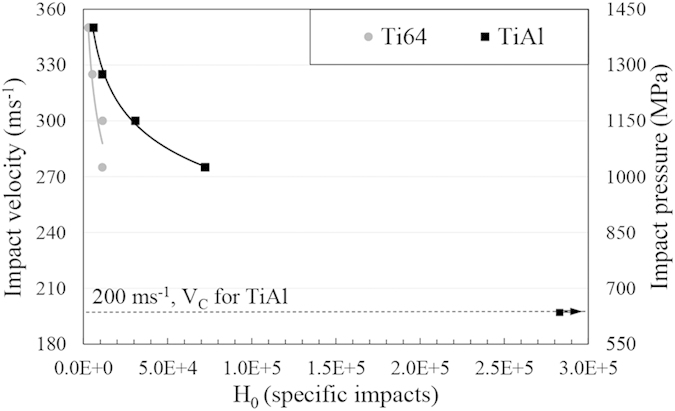
The dependency of Ti64 and TiAl incubation period on the impact speeds when they are eroded by 603 μm water droplets. The arrow indicates the test performed at 200 ms^−1^ and caused no measurable erosion after 10^6^ impact per 1 mm^2^.

Figures 7 and 8 show the relation between the incubation periods of Ti64 and TiAl alloys and impact speeds/impact pressures. The plotted curves show similar behaviour to fatigue S-N curves. To find the erosion endurance of TiAl eroded by 464 μm water droplets, the range of impact speeds was widened and erosion experiment was carried out at speed down to 250 ms^−1^. At this speed, no mass loss was observed after one million droplet impacts per 1 mm^2^ corresponding to 240,000 specific impacts. Hence, 250 ms^−1^ was assigned as the threshold speed for TiAl when 464 μm droplets were utilized, shown by arrow in Fig. 7. Knowing *Vc* in case of 464 μm water droplets and using equation (4), the critical-velocity-constant of TiAl was calculated. Based on this constant and equation (4), we should be able to estimate *Vc* in case of using any droplets size for TiAl. To prove that, the threshold speed for 603 μm droplets were theoretically calculated and the corresponding experiment for that speed were carried out. The *Vc* for 603 μm was calculated to be 202 ms^−1^. Then, the erosion experiment was carried out at 200 ms^−1^ impact speed. No erosion damage was detected after one million impacts per 1 mm^2^ (280,000 specific impacts), shown in Fig. 8. Therefore, 200 ms^−1^ was assigned as threshold speed of TiAl when eroded by 603 μm water droplets. The experiment proved that equation (4) is applicable to find the threshold speed for TiAl alloy when subjected to water droplet erosion.

In contrast to TiAl, the critical-velocity-constant could not be calculated for Ti64 from the curve presented in Fig. 7. Indeed, the critical-velocity-constant is a function of target material and may be correlated to its mechanical properties. Since, Ti64 shows lower erosion resistance compared to TiAl and presents much shorter incubation periods, additional experiments corresponding to the lower impact speeds are essential to determine threshold speed. Therefore, it will be the subject of a future study.

**Figure 9:**
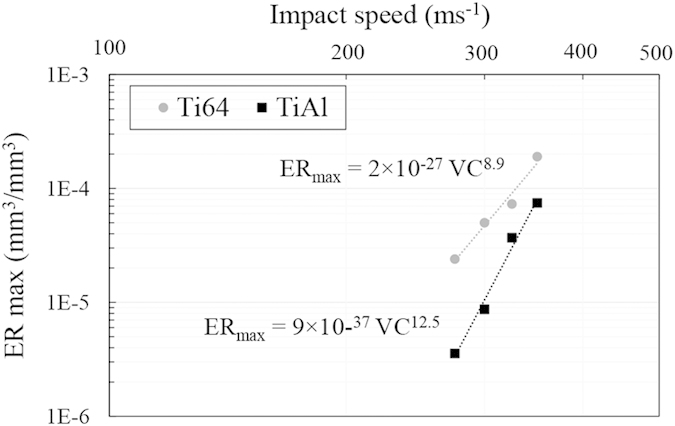
Dependency of maximum erosion rate on the impact speed for Ti64 and TiAl alloys eroded by 464 μm water droplets.

**Figure 10:**
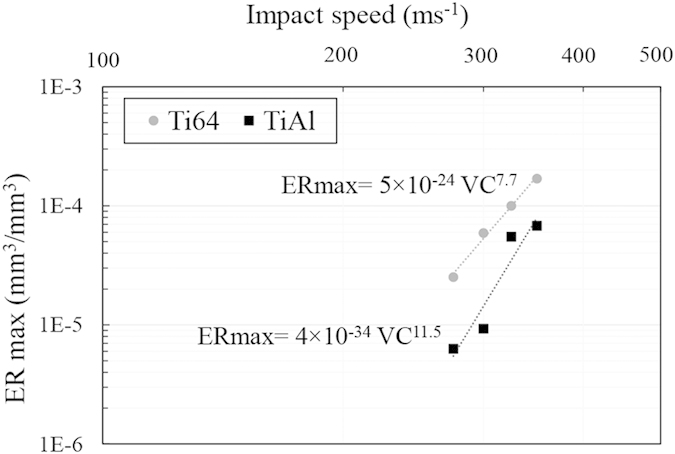
Dependency of maximum erosion rate on the impact speed for Ti64 and TiAl alloys eroded by 603 μm water droplets.

The maximum erosion rate versus impact speed for Ti64 and TiAl are presented in Figs 9 and 10. The experimental data are plotted in log-log graphs and fitted using a power law relationship. The slope of the fitting line is reported as the speed-dependency-exponent. In the case of the 464 μm droplets, it was found that the *ER*_*max*_ is related to the impact speed with 8.9 and 12.5 exponents for Ti64 and TiAl, respectively. However, for 603 μm droplets, it was found to be 7.7 and 11.5, respectively. Ti64 and TiAl are considered as erosion resistant alloys, but their material loss rates were increased notably with increasing the impact speed. TiAl erosion resistance drops significantly by increasing impact speed.

#### Comparison between TiAl and Ti64 WDE performances

**Figure 11:**
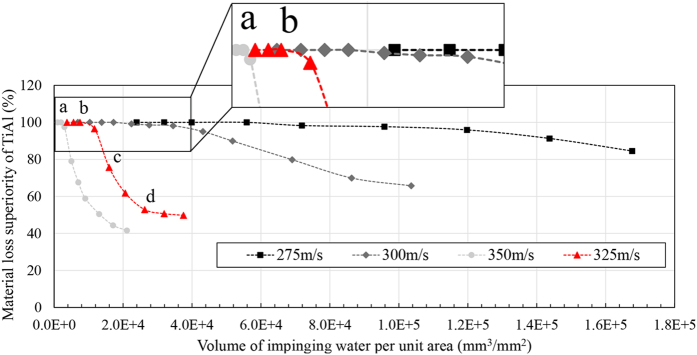
Material loss superiority of TiAl compared to Ti64 during the WDE test performed using 464 μm drops.

**Figure 12:**
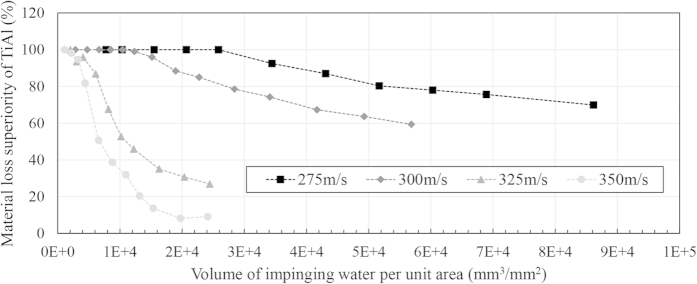
Material loss superiority of TiAl compared to Ti64 during the WDE test performed using 603 μm drops.

In order to compare the response of TiAl with that of Ti64 during the erosion test, the percentage of superiority at each interval versus volume of impinging water was plotted at different impact speeds. Figures 11 and 12 exhibit these graphs for 464 μm and 603 μm droplets.


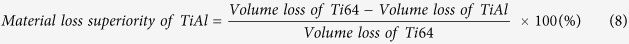


TiAl shows superior erosion resistance in all WDE test conditions used in this study. In the case of low impact speeds or small droplets, higher superiority can be seen. However, difference in erosion performance becomes less significant at high impact speeds or large water droplets. It seems that TiAl shows very high erosion resistance as the severity of the erosion conditions decreases. In order to understand these material loss superiority graphs, the curve corresponding to 325 ms^−1^ impact speed and 464 μm droplet size, shown by triangle markers in Fig. 11, is explained here. At the beginning of the experiment and during the incubation of Ti64 and TiAl, superiority is meaningless, zero divided by zero in the equation (8), and it is not reported in the graph. Thereby, the curves do not start from zero, shown in the magnified parts of Fig. 11. The beginning of the curve is once the material loss of Ti64 initiates (point a). This point represent 100 percent superiority of TiAl since it did not lose any material yet, but Ti64 did. The graph continues with 100% superiority until TiAl material loss initiates (point b). Further impacts lead to TiAl material loss and decrease in superiority from point b to point c. The reduction of erosion superiority is attributed to the different erosion stages experienced by the specimens. For instance, when Ti64 is undergoing a reduction in the erosion rate in the last stages of erosion (deceleration or terminal erosion rate), TiAl might still be in the second or third stages of erosion (acceleration or maximum erosion rate). The superiority curve keeps decreasing until it reaches a plateau (point d), when 30,000 mm^3^ of water impacted 1 mm^2^ of target surface. This plateau is detected because both specimens reach their terminal erosion stages.

It is expected to see such plateau of superiority for all conditions when both specimens reach their terminal erosion rate stage. This plateau was observed for some of the experiments performed mainly at severe conditions such as 48 percent for 464 μm and 325 m/s or 12 percent for 603 μm and 350 m/s. However, it was not revealed for the experiments performed at low impact speeds because the terminal stage of erosion for TiAl was not reached in these conditions. This type of representation is very helpful for the erosion comparison of two bulk materials especially their behavior at the later stages. The value of plateau can be reported as the superiority of TiAl at later stages of service compared to Ti64.

### Microscopic observation of erosion damage

**Figure 13:**
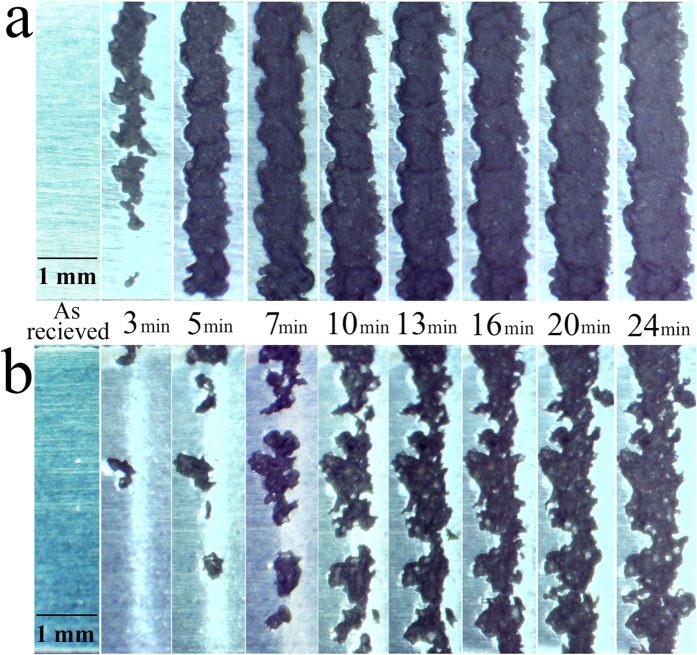
Macrographs of erosion craters at different time intervals (mentioned between two images) for the erosion experiments performed at 325 ms^−1^ impact speed and 603 μm droplet size: (a) Ti64 (b) TiAl.

**Figure 14:**
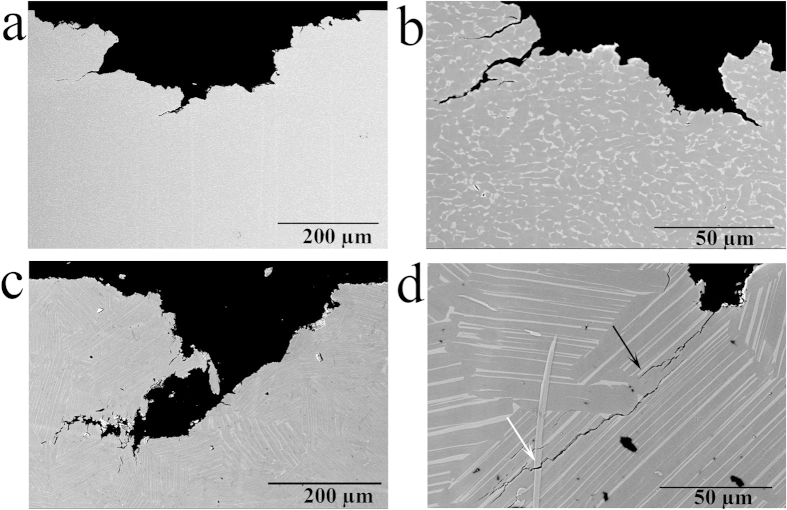
Cross sectional SEM micrographs of eroded (a,b) Ti64 and (c,d) TiAl: (a,c) Erosion pits at early stages, (b,d) influence of local microstructure on cracking behaviour.

As mentioned, the WDE tests were interrupted at different time intervals to weigh the coupons and record the material loss. Moreover, the areas exposed to the impacts were observed under an optical microscope to document the erosion features during the test. As a result, the pitting at different positions, the growth of eroded regions, their merging, formation of craters and grooves were recorded for the whole WDE test. For instance, the eroded Ti64 and TiAl tested at impact speed of 325 ms^−1^ and droplet size of 603 μm are illustrated in Fig. 13. Pitting and erosion damages can be seen on Ti64 earlier than TiAl, which corresponds to the observed longer incubation for TiAl. The material loss rate for both alloys increased after the formation of initial pits. However, there is a considerable difference between Ti64 and TiAl erosion progression. Ti64 lost material from all area exposed to the erosion. The pits merged with one another and formed complete erosion crater after three minutes erosion. Nevertheless, in the case of TiAl, instead of fast pits coalescence and having a complete erosion line, the initially damaged areas mostly became deeper and deeper by water droplet impacts. Then, as the formed pits enlarged by further impacts, they started to merge. The formation of deeper pits for TiAl compared to Ti64 at early stages of erosion is shown in Fig. 14a,c. They demonstrate the cross sections of typical pits formed during the erosion. Moreover, the cracking behaviours with respect to the local microstructures are presented in Fig. 14b,d. In the case of Ti64, relatively small intergranular and transgranular cracks can be seen. However, the trasngranular cracking (combination of interlamellar and translamellar cracks) are dominant in the case of TiAl.

## Discussion

The water droplet erosion performance strongly depends on the erosion conditions and tests need to be done in representative conditions. In order to understand the erosion behaviour of any material or in the case of comparing the performances of two different alloys, different erosion parameters need to be evaluated. In this study, the maximum erosion rate and incubation period of Ti64 and TiAl alloys tested at different impact velocities and droplet sizes are compared. Furthermore, the erosion dependency of Ti64 and TiAl alloys on the impact speed is compared. The obtained results are discussed below.

The erosion performance of TiAl is superior compared to Ti64, as mentioned in the results section. Figures 5 and 6 illustrating the maximum erosion rates and incubation periods confirm the lower *ER*_*max*_ and longer *H*_*0*_ for TiAl. Higher erosion resistance can be explained by two parameters, mechanical properties and microstructure. The hardness, yield and ultimate strength, modulus of resilience and toughness are considered important mechanical properties that affect the erosion resistance. The hardness and strength of TiAl are higher than those of Ti64 (up to 20 percent). Thus, they are in accordance with the WDE theories suggesting that these are key mechanical properties to study water droplet erosion. In terms of energy absorption, it was claimed that the higher the ability to absorb energy, the higher the resistance to erosion^3^,^4^. Because the amount of energy transferred to the target from impacting water droplet was considered as an important cause of the erosion damage. Ti64 can absorb more energy than TiAl during elastic deformation, since its modulus of resilience is higher than that of TiAl (shown in Table 3). But, Ti64 showed worse erosion resistance compared to TiAl and it means high resilience does not indicate high erosion resistance. Since the energy exerted on the target material upon water droplet impact might exceed the elastic energy, it may appear more appropriate to consider total absorbed energy before fracture, which is the toughness^11^. Toughness is roughly approximated by the area under σ-ε curve of each material. It is 450,000 kJm^−3^ for Ti64^24^ and 420,000 kJm^−3^ for TiAl^25^ in the case of static compression condition. Although Ti64 possess higher toughness than TiAl, its water erosion resistance is worse. Thereby, neither static resilience nor toughness can be assumed as a key mechanical property to address erosion behavior of Ti64 and TiAl. Since there are high speed impacts and as a result high strain rates, the dynamic mechanical properties of TiAl and Ti64 should also be considered. The higher the strain rate, the higher the strength and the energy required to fracture both alloys^26^,^27^. However, in the case of TiAl with lamellar microstructure, the influence of strain rate on the mechanical properties is significant^27^. Applying the load at a high strain rate results in much slower crack initiation and propagation rates^27^,^28^,^29^. Accordingly, the lamellar TiAl alloy shows much higher strength under dynamic loading conditions than Ti64^26^,^27^. They can be approximated around 1400 MPa for Ti64 and 2000 MPa for TiAl. For this reason and due to the nature of the repetitive loading during WDE, TiAl outperformed Ti64.

**Figure 15:**
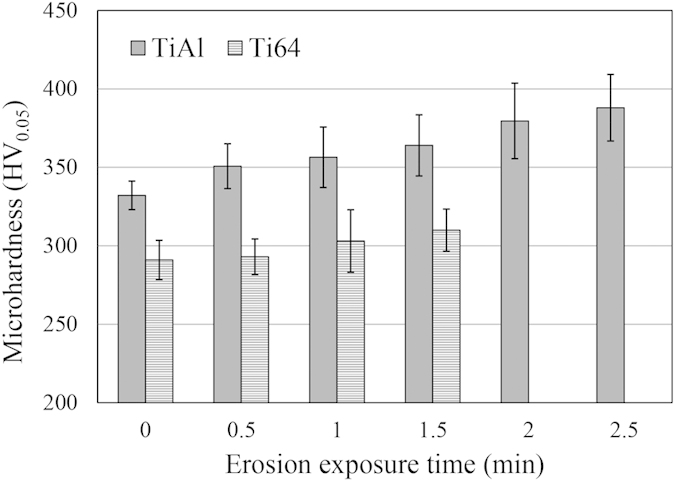
Surface hardness of TiAl and Ti64 presented in 0.5 min intervals during the incubation of erosion experiment performed at 350 ms^−1^ using 464 μm droplets.

Furthermore, the superior cavitation erosion resistance of TiAl was attributed to high strain hardenability of this alloy by Howard *et al.*^21^. Here, hardenability means that a higher value of stress would be required to cause deformation or failure after each collision. Similar condition might be experienced during water droplet erosion. To study the strain hardening of these alloys, their hardness were measured in 0.5 min intervals during the incubation of water droplet erosion (i.e. 1.5 min for Ti64 and 2.5 min for TiAl). Figure 15 shows that TiAl hardness increases by 11 and 17 percent after being subjected to repetitive water droplet impacts for 1.5 and 2.5 min, respectively. However, Ti64 hardness increases by 6 percent only after 1.5 min. Such strain hardening observed for TiAl can be assumed as an important reason for its superior WDE resistance especially for its long incubation period and lower maximum erosion rate.

It was proposed by Heymann^11^ that an empirical combination of strength and modulus of elasticity, σ_u_^2^E, is the most corresponding mechanical property to the erosion performance. Thus


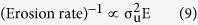


In order to address the influence of strain hardening coefficient in equation (9), we assume that there is a linear behaviour for the stress-strain relationship of materials up to yield point (Hooke’s law). Also, it is supposed that their plastic deformation (from the onset of the plastic deformation to the tensile strength point) are governed by a power equation (Hollomon’s law). By equating the stress-strain relationship for elastic and plastic regions at the yield point,


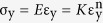


the modulus of elasticity can be written as:


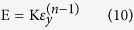


Where K is material’s constant, ε_y_ is the strain at the yield point and n is strain hardening coefficient. Therefore, equation (9) can be written as


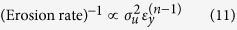


TiAl possess higher values for σ_u_ and n than Ti64 and it is in accordance with the water droplet erosion results, where TiAl outperformed Ti64. Hence, hardness, ultimate strength and strain hardening coefficient are confirmed to be the key mechanical properties influencing erosion performance.

According to the reported fatigue like mechanism of water droplet erosion^1^,^5^, the cracks’ propagation rate and their preferred direction for the studied materials would be important parameters. The effect of the microstructure is a considerable factor, especially in the case of TiAl^20^,^29^. The near fully lamellar TiAl, used in this study, shows superior crack growth resistance compared to the other types of TiAl^22^. This was attributed to the beneficial shielding effects of crack blunting or deflection because of different colony orientations, shown by black arrow in Fig. 14d. Moreover, ligament bridging toughening is another reason for the high resistance of lamellar TiAl against crack growth^29^. This leads to crack growth deceleration and is shown by white arrow in Fig. 14d. Micro-cracking ahead of the tip of the main crack was also reported to delay the crack propagation especially in cyclic loading conditions^22^. Such micro-cracking ahead of one long crack can be seen in Fig. 14a. Although cracks deflection, bifurcation, and ligament bridging decelerate the cracks propagation within the lamellar microstructure, in a few cases relatively large cracks are observed along the craters edges, as can be seen in Fig. 14d. Such cracking may cause slow and infrequent detachment of relatively large fragments of TiAl during the erosion. This is not the case for Ti64. Indeed, the local microstructure of Ti64 with small and equiaxed α grains leads to faster cracks coalescence and subsequent material loss. Therefore, fast and frequent detachment of small fragments would be a dominant damage mechanism for Ti64. This corresponds to the lower erosion resistance of Ti64 compared to TiAl.

Unlike Ti64 alloy, the difference in *ER*_*max*_ of TiAl alloy between tests done at the impact speeds of 300 ms^−1^ and 325 ms^−1^ is considerably high, if compared to the *ER*_*max*_ differences for other speed intervals. It was more evident, when 603 μm droplets were used in the test. In fact, *ER*_*max*_ increased more than 6 times when the impact speed increased from 300 ms^−1^ to 325 ms^−1^. It can be attributed to a potential level of imposed stress causing significantly high crack propagation rates. Showing this type of critical stresses under dynamic loading is a known behaviour for intermetallics^22^. Furthermore, in most of WDE studies the water properties were assumed to be constant, neglecting its viscoelasticity. In light of the results in this paper and considering the high strain rate during the impact, a hypothesis can be made to explain the sudden increase in erosion rates for materials at some specific speeds, in terms of the viscoelastic properties of water and their dependency on impact speed. It is known that for viscoelastic liquids, the speed of impact can determine their response^30^. Thus, it can affect the amount of energy transferred to the material, and the amount causing the deformation of the water droplet itself. It was not proved experimentally; however, this point can be tackled in a future work.

In spite of different available equations for erosion-impact speed relationship, the experimental data was fitted using equation (1) as discussed in the results section. The speed exponent was mainly reported between 5 to 7 for metals^6^,^9^; however, it was found to be more than 7 for Ti64 in this study. In previous works, similar inconsistency was observed, Table 1^6^,^7^,^9^,^10^. The main reasons for such a difference in values of the speed exponent, can be attributed to the different WDE rigs, different erosion test conditions and different erosion indicators on which the influence of impact velocity has been investigated. For instance, the influence of the impinging water characteristics (shape and size) on the speed dependency exponent were neglected in most of previous works since they generalized the speed dependency exponent^6^,^7^,^10^. The erosion severity including impact pressures and duration of each impact pulse is a function of both the speed and the water droplet size, Table 2. Hence, the speed exponents acquired from the tests performed using different conditions for the impinging water droplets, are expected to have different values. Since there is no clear physical explanation for the observed trends and the determined speed exponents, the influence of the water droplet conditions on the erosion rate-impact speed relationship needs to be studied experimentally, while keeping other parameters constant. In the current study, the experiments were carried out using two different droplet sizes. The speed exponents were found to be 8.9 and 7.7 for Ti64 in the case of 464 and 603 μm droplets, respectively. This observed difference confirms that the speed exponent is function of droplet size, or generally speaking the “impact conditions”. It verifies Heymann’s conclusion that the presented erosion-speed relationship using a certain exponent, equation (1), would be valid over limited speed ranges^4^.

Furthermore, the properties of target material affect these parameters. Higher speed exponent was reported for the erosion of ceramics compared to metals^4^,^10^. Larger exponent would imply a higher tendency for brittle failure, and a higher transfer of impact energy through the interface between the droplet and the target material^31^. There is a notable difference between Ti64 as a metal and TiAl as an intermetallic in terms of their erosion dependency on the impact speed. The erosion rate is linked to the crack propagation, and it is the main reason for material loss in terminal stages of the water droplet erosion damage. For intermetallics, especially TiAl, the crack propagation rate is extremely sensitive to the stress intensity. It was proved by having a large exponent in the Paris law^22^, which is 5 to 10 times larger than typical values of metallic systems. Since TiAl tends to act in a more brittle manner due to its high sensitivity to speed increase, it has higher speed exponent than that of Ti64 which is in accord with the current results.

As mentioned earlier, critical or threshold speeds for the erosion damage were estimated for TiAl. This approach was used by researchers in the field of cavitation erosion or water jet erosion^6^,^8^,^32^. The attempt was to relate the erosion endurance to the fatigue limit. Thirwvengadam^6^ compared the water erosion endurance with the fatigue strength (obtained from magnetostriction oscillator setup for fatigue test) for stainless steel and Ti64. The erosion endurance was found to be half and one third of the fatigue strength for Ti64 and stainless steel, respectively. This considerable difference was correlated to the local fatigue failure caused by water erosion which could not be represented by the used fatigue setup. In fact, the considerable difference in endurance levels for materials subjected to conventional fatigue and WDE would confirm the divergence in material’s strength for both loading conditions, despite their similarity in terms of cyclic loading. The drawback of Thirwvengadam’s work was neglecting the variation of the critical speed. This speed was found to be a function of droplet size based on equation (4). The product of the droplet diameter and *V*_*C*_^2^ being critical-velocity-constant, was proposed and confirmed mainly for metals^4^. This work affirms the validity of this equation in representing erosion for an intermetallic as TiAl, seen in Figs 7 and 8. It is noteworthy that the droplet size distributions presented in the result section confirm the accuracy of equation (4) in the case of water droplet erosion damage. Here, the volume fraction distributions show that for both nozzles large volume of water (more than 75 percent) impinging the target are droplets that their diameter standard deviation from the mean value is less than 5 percent.

In conclusion, the superior erosion resistance of nearly fully lamellar TiAl compared to Ti64 is observed at all tested conditions. However, the magnitude of its superiority depends on the impact speed and droplet size. The less the severity of erosion test, the higher the superiority of TiAl erosion resistance compared to Ti64. Such superiority could be attributed to the higher hardness, strength, modulus of elasticity, hardenability and lamellar microstructure. Indeed, the randomly oriented TiAl colonies with fine lamellar microstructure result in relatively low crack growth rate improving erosion resistance. Furthermore, the maximum erosion rate and impact speed relationship for both tested materials, a metallic and an intermetallic alloys, showed a linear trend on the logarithmic scale. For Ti64, the speed exponent was found to be 8.9 and 7.7 for 464 and 603 μm droplets, respectively. In the case of TiAl alloy, it was found to be 12.5 and 11.5 for 464 and 603 μm droplets, respectively. The observed exponents for Ti64 are higher than the reported values in the literature, 5 to 7. This difference is attributed to the erosion test conditions and different approaches used to derive the erosion rate. Finally, the threshold speed was found to be function of the impinging droplet diameter so that the product of the droplet diameter and *V*_*C*_^*2*^ is constant. Using such equation, the threshold speeds for the TiAl alloy eroded by 464 and 603 μm water droplets were determined to be around 250 ms^−1^ and 200 ms^−1^, respectively.

## Materials and Methods

### Materials

**Figure 16:**
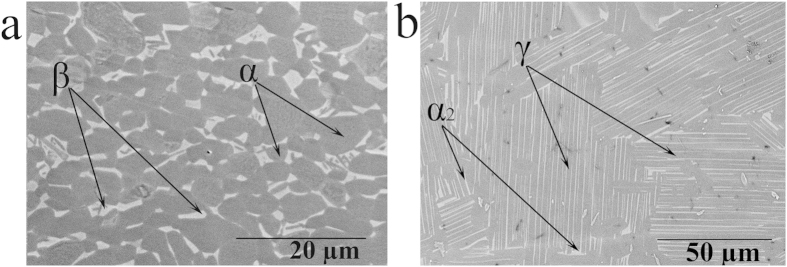
SEM micrographs of (a) annealed Ti64 (b) HIP TiAl.

An annealed Ti6Al4V sheet and a heat treated Ti45Al2Nb2Mn0.8TiB_2_ (45-2-2XD) plate were received from Titanium Industries Inc. and Rolls-Royce Canada Ltd, respectively. They are referred to as Ti64 and TiAl in this paper. Erosion specimens (23 mm × 8 mm × 3 mm) were prepared from these two alloys. The target surfaces were ground using SiC grinding papers of 600 grit size. The surface roughness of erosion coupons was kept as similar as possible to minimize its influence on the erosion behaviours. The received Ti64 contains α and β phases with a homogenized microstructure as shown in Fig. 16a. The studied TiAl alloy was received as a plate with special treatment (hot isostatic pressed and then heat treated). The microstructure of the TiAl alloy shows near fully lamellar structure of α_2_ phase in γ phase matrix, as shown in the Fig. 16b. The density and mechanical properties of these alloys including their young’s modulus, yield strength, and modulus of resilience are listed in Table 3. The young’s modulus was taken from the literature^22^,^24^,^33^. The yield strengths values were inferred from the hardness measured in this work. They are approximately 1/3 times the Vickers hardness^34^. Modulus of resilience was derived from the yield strength and Young’s modulus. In this work, erosion performance is explained based on the mechanical properties and microstructures.

### Water droplet erosion test

**Figure 17:**
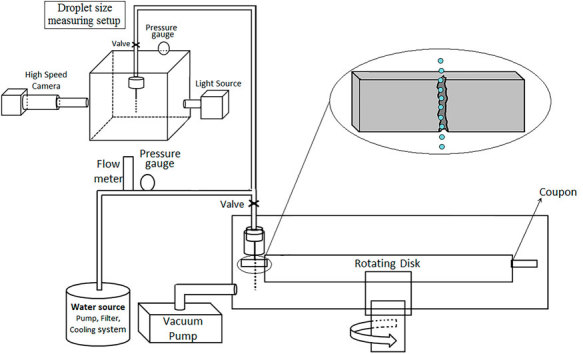
Schematic of the water droplet erosion rig and droplet size measuring setup.

The water droplet erosion test was performed using a rotating disk erosion rig with controlled conditions. It was designed based on the ASTM international G73 standard^13^. It provides simulated impingement conditions between high speed rotating blades and liquid droplets for erosion studies. A schematic of the erosion rig including water droplet generation system is presented in Fig. 17. The rotating disk can reach a maximum linear speed of 500 ms^−1^. The erosion test coupons should be mounted on the disk and based on the desired impact speed, the rotation speed would be set. The coupons mounted on the rotating disk are subjected to the water droplets with an impact angle of 90°, as can be seen in Fig. 17. In order to produce the water droplets, distilled water is pumped into the droplet generation system after passing through 5 micron filters. The water generation system includes a flow meter, a pressure gauge and a nozzle. The pressure of the pumped water and its flow were optimized to have water droplets in desired size and narrow size distribution. The nozzle is shielded against the turbulence occurring inside the chamber to ensure the straightness of water stream with minimum deviation and droplet distortion till impact. To acquire the droplet size distribution, a transparent box, shown in Fig. 17, simulating the erosion chamber in terms of its pressure and the water droplets conditions was used. The water droplet diameters were measured in the box when the same water droplet generation system, nozzle, back pressure and flow rate were utilized. The falling droplets were monitored using a high speed camera and the diameters of 200 droplets were measured. The erosion test results are presented in the form of cumulative material loss versus cumulative exposure. Accordingly, the erosion tests were divided into several time intervals, in order to measure the mass loss per interval. After each interval, coupons were weighed five times. Their average weight was recorded and the difference from the initial mass was taken as the cumulative mass loss. The volumetric material loss was derived using the measured eroded mass and the density of the tested alloys. To make the erosion results consistent and following the ASTM standard, the obtained volume loss was normalized by the area affected by water droplets. Such area was measured from the optical micrographs recorded at the beginning of the maximum erosion rate stage. It is assumed to be the average of damaged surface area during the whole erosion experiment. Other erosion characteristics were obtained from these water erosion graphs. Since all the erosion results are derived from mass loss, a high precision (0.1 mg) balance was used. The maximum observed standard deviation was 0.2 mg, which is less than 2% of the average measured mass loss after each interval.

The erosion curves were plotted according to the ASTM G73 standard^13^. The erosion performance variables, incubation period (*H*_*0*_) and maximum erosion rate (*ER*_*max*_), were determined from the graphs. Since quantitative comparison among different impingement conditions is one of the goals of this study, rationalized and dimensionless values for erosion rate and erosion time were utilized, based on the ASTM standard. Therefore, specific impact and rationalized erosion rate are defined^13^ as:


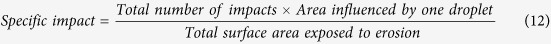



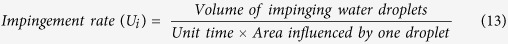



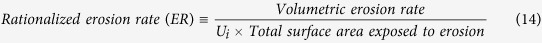


According to the ASTM G73 standard^13^, straight line best fit for the maximum slope points was plotted and its slope was considered as *ER*_*max*_. The incubation period *(H*_*0*_) was determined as the intersection of the fitted line with the X-axis.

**Figure 18:**
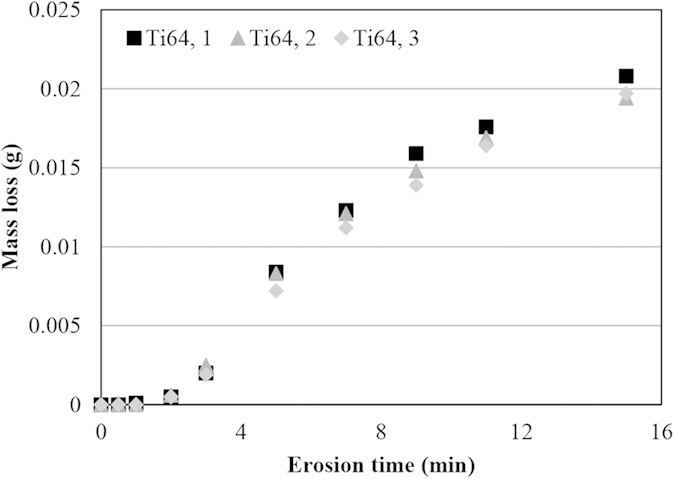
Erosion results of three Ti64 coupons tested at 350 ms^−1^ impact speed and 464 μm droplet size showing repeatable experimental results.

As explained earlier, the threshold speed corresponds to the impact speed below which a very long incubation period can be seen. To determine the threshold, this long incubation needs to be defined. In this work, *V*_*C*_ corresponds to the speed at which one million droplets impacts per 1 mm^2^ do not cause measurable mass loss. It is noteworthy that 25 ms^−1^ is the used interval in this work to study the influence of impact speed. Hence, the real critical impact speed would be between *V*_*C*_ (the speed at which one million droplets impacts per 1 mm^2^ do not cause measurable mass loss) and *V*_*C*_ + *25* (the speed at which one million droplets impacts per 1 mm^2^ cause measurable mass loss). Here, *V*_*C*_ which is the minimum of this range is reported as the critical impact speed for safety measures. An important aspect for experimental work is the repeatability of the tests. Therefore, the following experimental conditions were monitored and their repeatability was verified using sensors and gauges available on the test setup: stability of speed (rpm), stability of vacuum, and vibration. In order to ensure the repeatability and reliability of WDE test results, three specimens from annealed Ti64 alloy were prepared. The material and its properties are assumed to be identical. Three erosion tests were performed at the same conditions, 350 ms^−1^ impact speed and 464 μm droplet size. The results are presented in Fig. 18. They are consistent; however, some deviations can be seen. Similar deviations were reported in the literature^9^. The degree of deviation increases with the erosion time and higher deviation can be seen at the last stages. Erosion initiation and material loss is a function of coupon surface quality^1^,^13^,^35^. There is less deviation at the initial stages because of the preparation of the coupons, which leads to similar surface conditions. It is not the case for the later stages, since the formation of craters change the surface topography with same degree of variability and lead to different hydrodynamic loading on the target surface. The maximum standard deviation of mass loss in each interval was observed as 1 mg among the replicas, even at the later erosion stages. The coupon resulting surface topography, impurities in the water, accuracy of the used balance and microstructural variations might be the reasons for such differences. In the current study, the influence of impingement speed on the erosion behaviour of Ti64 and TiAl is studied. Four different impact speeds, 275, 300, 325, 350 ms^−1^, and two different droplet sizes were investigated as the erosion test conditions. Nozzle 1 and 2 were used to generate different droplet sizes of 464 μm and 603 μm, respectively.

### Imaging techniques

To document the microstructures of the as-received materials, a Hitachi S-3400N Scanning Electron Microscope (SEM) equipped with a backscattered electron detector was used. During the erosion experiment and after each time interval, the erosion craters were observed using an optical microscope MEIJI Techno IM7100 and their images were recorded. These optical images provide general idea of how erosion damage initiates, progresses and forms a complete erosion line.

### Hardness measurements

Vickers hardness tester was used to measure the surface hardness of Ti64 and TiAl specimens. Five indentations were performed (10 kg load) at different positions on the test samples and their average value was taken as the hardness. In order to compare the mechanical behaviour of tested specimens, their yield strength values were inferred from their measured hardness.

## Additional Information

**How to cite this article**: Mahdipoor, M.S. *et al.* Influence of impact speed on water droplet erosion of TiAl compared with Ti6Al4V. *Sci. Rep.* **5**, 14182; doi: 10.1038/srep14182 (2015).

## Figures and Tables

**Table 1:** The impact speed exponent in equation (1) found in different water erosion investigations.

Authors	Tested material	Range of impact speeds (ms^−1^)	Impinging water conditions	ER representation	Speed exponent range (n)
Thiruvengadam *et al.*^6^	Al, Ni, 316 Stainless Steel, Ti6Al4V	90–250	800 μm, water jet, rotating arm	Maximum material loss rate, cc(hr)^−1^	5–7
Oka *et al.*^7^	Stainless steel, TiN coated steel, Sprayed cermet coating	60–300	45 to 130 μm, water droplet, spraying water droplets on a stationary target	Erosion damage rate, mm^3^(kg)^−1^	3–5.5
Hackworth^10^	Zinc sulfide (for Infrared window application)	200–350	700 to 1800 μ m, water droplet (rain erosion), rotating arm	Transmittance loss^*^ rate (not material loss), % loss (min)^−1^	9–14
Ahmad *et al.*^9^	Stainless steel, Ti6Al4V	350–580	100–350 μm, spraying water droplet, rotating arm	Erosion resistance (reciprocal of average material loss rate) at 50 hours erosion, sm^−1^	3.8–5.3
Tsubouchi *et al.*^12^	Stainless steel	450–630	50 μm, water droplet, rotating arm	Average material loss rate at 30 hours erosion, mm(h)^−1^	5

*Since the rain erosion of materials for infrared window application was studied, the transmittance loss with respect to the original transparency was considered.

**Table 2:** Impact pressure and time duration of one impact pulse calculated for different erosion conditions based on equations (6) and (7).

Impingement conditions		Impact pressure (MPa)	Duration of the impact pulse (μs)
Droplet size (μm)	Impact speed (ms^−1^)		
464	275	1032	0.0234
	300	1148	0.0243
	325	1268	0.0251
	350	1392	0.0258
603	275	1032	0.0307
	300	1148	0.0319
	325	1268	0.0330
	350	1392	0.0339

**Table 3:** Mechanical properties of Ti64 and TiAl* 19,22,24,26,33,34.

	Density (g/cm^3^)	Young’s Modulus (GPa)	Yield Strength (MPa)	Modulus of Resilience, σy^2^ (2E)^−1^ (kJm^−3^)
Ti64	4.42	113	993	4.36
TiAl	4.00	160	1120	3.92

However, the most corresponding ones are presented here.

^*^Mechanical properties of TiAl may vary with microstructure.
